# Bone stress injuries in athletics (track and field) championships: findings from a prospective injury surveillance conducted across 24 international championships with 29,147 registered athletes

**DOI:** 10.1186/s13102-024-00955-w

**Published:** 2024-08-15

**Authors:** Tim Hoenig, Adam S. Tenforde, Karsten Hollander, Astrid Junge, Pedro Branco, Anders Vinther, Pascal Edouard

**Affiliations:** 1 Department of Trauma and Orthopaedic Surgery, University Medical Center Hamburg-Eppendorf, Martinistraße 52, 20251 Hamburg, Germany; 2 Department of Physical Medicine and Rehabilitation, Spaulding Rehabilitation Hospital, Harvard Medical School, Charlestown, MA 02129 USA; 3 Institute of Interdisciplinary Exercise Science and Sports Medicine, MSH Medical School Hamburg, Hamburg, Germany; 4 Center for Health in Performing Arts, MSH Medical School Hamburg, Hamburg, Germany; 5 European Athletics Medical & Anti Doping Commission, European Athletics Association (EAA), Lausanne, Switzerland; 6 Department of Physiotherapy and Occupational Therapy, Copenhagen University Hospital, Herlev and Gentofte, Copenhagen, Denmark; 7 Hospital Secretariat and Communication, Research, Copenhagen University Hospital, Herlev and Gentofte, Copenhagen, Denmark; 8 Inter-university Laboratory of Human Movement Science (LIBM EA 7424), University of Lyon, University Jean Monnet, Saint Etienne, F-42023 France; 9 Department of Clinical and Exercise Physiology, Sports Medicine Unit, University Hospital of Saint-Etienne, Faculty of Medicine, Saint-Etienne, France

**Keywords:** Bone, Fractures, Stress, Injury Surveillance, Prevention, Epidemiology, Track and Field

## Abstract

**Background:**

Athletics (track and field) athletes are prone to develop bone stress injuries (BSIs) but epidemiological data on BSIs from top-level sports events are scarce.

**Objective:**

To describe the incidence and characteristics of BSIs during 24 international athletics championships held from 2007 to 2023.

**Methods:**

BSI-related data were prospectively collected during 24 international athletics championships, including the Olympic Games (*n* = 3), World Outdoor Championships (*n* = 4), European Outdoor Championships (*n* = 6), World Indoor Championships (*n* = 3) and European Indoor Championships (*n* = 8). Descriptive and comparative statistics were used to assess the epidemiological characteristics of BSIs.

**Results:**

BSIs accounted for 1.5% of all reported injuries (*n* = 36; 1.2 per 1000 registered athletes (95%CI 0.8 to 1.6)). No significant difference of BSI incidence was detected between female (2.0 per 1000 athletes (95%CI: 0.9 to 2.3)) and male athletes (0.9 per 1000 athletes (95%CI: 0.4 to 1.4)) (relative risk (RR) = 1.73, 95%CI: 0.88 to 3.40). BSI incidence was significantly higher during outdoor championships (1.6 per 1000 registered athletes (95%CI: 1.0 to 2.1)) as compared to indoor championships (0.2 per 1000 registered athletes (95%CI: 0.0 to 0.5)) (RR = 10.4, 95%CI: 1.43 to 76.0). Most BSIs were sustained in the foot (*n* = 50%) or leg (*n* = 33%). BSIs were reported in athletes participating in endurance disciplines (52.8%) or in explosive disciplines (47.2%).

**Conclusions:**

BSIs represent a small portion of injuries sustained during international athletics championships. Collective results suggest that injury rates are higher in outdoor competitions as compared to indoor competitions. The most common injury locations comprise the foot and leg.

**Clinical Trial Number:**

Not applicable.

## Introduction

Bone stress injuries (BSIs) are a form of overuse injury in athletes that can result in long-term absence from sports participation [1]. The underlying mechanism of BSI is the accumulation of bony microdamage [2–4]. Due to the absence of a single traumatic event in many cases, BSIs are often associated with a delayed time to diagnosis [4]. Failure to make a correct diagnosis may result in the progression to a full fracture with prolonged time for return-to-sport and/or need for surgery [2, 5, 6].

Athletics (track and field) athletes are among those with the highest risk of BSI [1]. A 12-month prospective study reported an incidence rate of 20% over the course of one season [7]. Women have higher injury rates than men [8], and women who participate in cross-country running or outdoor athletics events were among those with the highest rates of BSIs [9]. This finding is supported by prior work during international athletics championships suggesting that BSIs are less common in male than female athletes (RR = 0.32 (95%CI: 0.12 to 0.81)) [10]. BSIs that can affect participation in competitions are of interest as attendance in international championships is the most important season goal of elite athletes. Previous reports on the epidemiology of BSIs during the summer Olympics in Rio de Janeiro 2016 [11] and Tokyo 2020 [12] reported that half of all BSIs occurred in athletics. General injury rates and characteristics during international athletics championships have been previously reported, but mainly with a focus on soft tissue injuries [10,13, 14, 15]. To build a framework for the treatment and management of BSIs, it is necessary in the first instance to have a detailed knowledge of the underlying epidemiological aspects. Despite knowledge that those who compete in elite athletics may be at elevated risk for BSIs, clear evidence about epidemiology of BSI among top-level athletes is still missing. Understanding the characteristics of those who sustain BSIs during athletic disciplines may guide sports medicine clinicians to detect BSIs and aid in the on-the-field evaluation of injured athletes.

The purpose of this study was to describe the incidence and characteristics of BSIs during 24 top-level international athletic championships and explore differences between genders and types of competition.

## Methods

The present study is part of a broader prospective injury surveillance program in athletics [15]. Its study design has been previously described in detail [15–17]. Data collection was based on standardized criteria and injury definitions [15–17]. Saint-Etienne University Hospital Ethics Committee approved this study protocol (Institutional Review Board: IORG0007394; IRBN742020/CHUSTE).

Injury data were collected during 24 international athletics championships (Table 1). Injuries were identified by using a reporting system. Daily reports of new injuries were completed by the medical staff of the national teams (including physiotherapists and physicians) and/or by physicians of the local organising committee (LOC). Each were instructed to report “all musculoskeletal injuries (traumatic and overuse) newly incurred during competition or training regardless of the consequences with respect to the athlete’s absence from competition or training” [15–18]. All injuries were reported anonymously. Injuries were classified by location, type, cause, severity, circumstance and discipline (event) as described in the consensus statement for epidemiological studies in athletics [18]. In the present study, we analysed all *bone stress injuries / stress fractures* that were reported to the database. These injuries comprised, by definition, all bone injuries due to overuse as compared to traumatic bone fractures [2]. Each BSI was classified by anatomy, severity and athletics discipline.

**Table 1:** International championships included in the injury surveillance

Championships	
World Outdoor Championships (WOC) 2007 [16], 2009 [20], 2011 [21], 2013 [22] Olympic Games (OG) 2008 [23], 2012 [24], and 2016 [25] European Outdoor Championships (EOC) 2010, 2012 [26], 2014, 2016, 2018, 2022 World Indoor Championships (WIC) 2008, 2010, 2014 European Indoor Championships (EIC) 2009, 2011 [27], 2013 [28], 2015, 2017, 2019, 2021 and 2023	

Using similar criteria as Edouard et al. [19], the total number of participating athletes was determined by using the athletes registrations at each of the 24 championships from the International Association of Athletics Federations (IAAF, now World Athletics) or European Athletics (EA) for each championship (i.e., an athlete counted for more than one championship if he/she registered for multiple championships).

The primary outcome of BSIs sustained during championships was reported in total number, percentage of all injuries, and BSIs per 1000 registered athletes (with 95% confidence intervals). All data were reported by sex, discipline and type of championships (outdoor vs. indoor championships). In addition, differences in distribution between female and male athletes, and between outdoor and indoor championships, were analysed. Chi [2]-tests or Fisher’s exact test were used where appropriate. Significance was set at *p* < 0.05.

## Results

A total of 29,147 (13,506 female and 15,641 male) athletes were registered for the 24 international athletics championships. Overall, 2,362 injuries were reported and 36 were classified as BSIs, representing 1.5% of all injuries (Table 2). The incidence rate was 1.2 per 1000 registered athletes (95%CI 0.8 to 1.6). National medical team participation, athletes’ coverage, response rate and injury data are presented in Table 2.

**Table 2:** National medical team participation, athletes’ coverage and response rate, number of registered athletes, number of all injuries and bone stress injuries and number of bone stress injuries per 1000 registered athletes for 24 international athletics championships from 2007–2023. WOC, World Outdoor championships; OG, Olympic games; EOC, European Outdoor championships; WIC, world indoor championships; EIC, European indoor championships

Championships	Total number of days	Total number of countries	Percentage of countries participating in the injury data collection	Percentage of countries participating in the injury data collection among countries with registered medical teams	Percentage of athletes covered by the participating medical teams	Response rate (%) by participating medical teams	Number of registered athletes	Number of registered female athletes	Number of registered male athletes	Number of all injuries	Number of bone stress injuries	Percentage of bone stress injuries	Number of bone injuries per 1000 registered athletes	Number of bone stress injuries in female athletes	Number of bone stress injuries in male athletes	Number of bone stress injuries in female athletes per 1000 registered female athletes	Number of bone stress injuries in male athletes per 1000 registered male athletes
WOC 2007	9	198	25.3	90.9	71.1	74.0	1807	854	953	205	5	2.4	2.8	4	1	4.7	1.0
WOC 2009	9	200	23.5	111.9	74.9	89.8	1896	855	1041	262	5	1.9	2.6	2	3	2.3	2.9
WOC 2011	9	199	30.7	107.0	81.3	93.8	1741	811	930	225	2	0.9	1.1	2	0	2.5	0.0
WOC 2013	9	203	22.2	63.4	74.2	89.9	1784	784	1000	180	4	2.2	2.2	3	1	3.8	1.0
OG 2008	9	204					2132	1001	1131	159	4	2.5	1.9	3	1	3.0	0.9
OG 2012	9	204					2079	991	1088	213	5	2.3	2.4	4	1	4.0	0.9
OG 2016	9	207					2367	1140	1227	158	0	0.0	0.0	0	0	0.0	0.0
EOC 2010	6	50	48.0	68.6	83.1	73.6	1371	609	762	51	0	0.0	0.0	0	0	0.0	0.0
EOC 2012	5	50	60.0	83.3	92.8	90.7	1352	607	745	126	0	0.0	0.0	0	0	0.0	0.0
EOC 2014	6	50	66.0	97.1	95.4	91.4	1439	652	787	141	4	2.8	2.8	1	3	1.5	3.8
EOC 2016	5	50	64.0	88.9	83.0	94.4	1469	719	750	90	1	1.1	0.7	0	1	0.0	1.3
EOC 2018	7	51	60.8	79.5	82.2	100.0	1570	742	828	98	5	5.1	3.2	2	3	2.7	3.6
EOC 2022	7	48	64.6	93.9	93.4	100.0	1540	735	805	78	0	0.0	0.0	0	0	0.0	0.0
WIC 2008	3	147	23.1	97.1	63.2	89.2	573	240	333	48	0	0.0	0.0	0	0	0.0	0.0
WIC 2010	3	142					583	249	334	32	0	0.0	0.0	0	0	0.0	0.0
WIC 2014	3	134	27.6	84.1	71.6	100.0	539	253	286	33	0	0.0	0.0	0	0	0.0	0.0
EIC 2009	3	45	62.2	90.3	89.7	85.7	568	252	316	35	0	0.0	0.0	0	0	0.0	0.0
EIC 2011	3	46	37.0	53.1	69.5	84.3	593	269	324	29	0	0.0	0.0	0	0	0.0	0.0
EIC 2013	3	47	61.7	100.0	91.5	100.0	577	257	320	60	1	1.7	1.7	1	0	3.9	0.0
EIC 2015	4	49	55.1	79.4	75.6	97.2	643	280	363	27	0	0.0	0.0	0	0	0.0	0.0
EIC 2017	3	49	65.3	94.1	92.7	96.9	561	279	282	19	0	0.0	0.0	0	0	0.0	0.0
EIC 2019	3	49	63.3	86.1	90.7	96.8	637	307	330	33	0	0.0	0.0	0	0	0.0	0.0
EIC 2021	4	47	48.9	92.0	92.6	96.7	733	328	405	29	0	0.0	0.0	0	0	0.0	0.0
EIC 2023	4	47	57.4	93.1	83.0	100.0	593	292	301	31	0	0.0	0.0	0	0	0.0	0.0
**Total outdoor championships**	**99**	**1714**	**46.5**	**88.4**	**83.1**	**89.8**	**22,547**	**10,500**	**12,047**	**1986**	**35**	**1.8**	**1.6**	**21**	**14**	**2.0**	**1.2**
**Total indoor championships**	**36**	**802**	**50.2**	**86.9**	**82.0**	**94.7**	**6600**	**3006**	**3594**	**376**	**1**	**0.3**	**0.2**	**1**	**0**	**0.3**	**0.0**
**TOTAL**	**135**	**2516**	**48.3**	**87.7**	**82.6**	**92.2**	**29,147**	**13,506**	**15,641**	**2362**	**36**	**1.5**	**1.2**	**22**	**14**	**1.6**	**0.9**

Most BSIs were sustained by female athletes (*n* = 22, 61.1%; 2.0 injuries per 1000 registered female athletes; 95%CI 0.9–2.3), while the remainder occurred in male athletes (*n* = 14, 38.9%; 0.9 injuries per 1000 registered male athletes; 95%CI 0.4–1.4). The incidence of BSIs was not significantly higher in female compared to male athletes (relative risk (RR) = 1.73, 95%CI 0.88–3.40).

Nearly all BSIs (*n* = 35, 97.2%) were sustained during outdoor international athletics championships (Table 2). The incidence rate was significantly higher in outdoor than in indoor championships (1.6 (95%CI: 1.0 to 2.1) vs. 0.2 (95%CI: 0.0 to 0.5) injuries per 1000 registered athletes; RR = 10.4, 95%CI: 1.43 to 76.0).

About half of all injuries were either reported in athletes participating in endurance disciplines (52.8%) or in explosive disciplines (47.2%). No BSIs were reported in throwing disciplines. The distribution of BSI location according to disciplines is presented in Table 3.

Most BSIs were sustained in the lower extremity (*n* = 33; 91.7%). The foot (50.0%) and the lower leg (33.3%) were the most frequent anatomical locations of injury (Table 3). Twenty BSIs (55.6%) were classified as severe injuries (estimated absence > 28 days), four as moderate injuries (estimated absence 7–28 days) and one as a minor injury (estimated absence < 7 days). Six BSIs were classified as no-time loss injuries. Time of absence was missing for five injuries.

**Table 3:** Distribution of bone stress injury location according to sex and disciplines during the 24 international athletics championships from 2007–2023

	Neck / cervical spine	Lumbar Spine	Pelvis / Sacrum	Wrist	Lower leg	Ankle	Foot	Total
**Total**	**1 (2.8%)**	**1 (2.8%)**	**1 (2.8%)**	**1 (2.8%)**	**12 (33.3%)**	**2 (5.6%)**	**18 (50.0%)**	**36 (100.0%)**
**Female athletes**	**1 (2.8%)**			**1 (2.8%)**	**10 (27.8%)**	**1 (2.8%)**	**9 (25.0%)**	**22 (61.1%)**
Sprints	1 (2.8%)				1 (2.8%)		2 (5.6%)	4 (11.1%)
Hurdles					1 (2.8%)		1 (2.8%)	2 (5.6%)
Jumps					1 (2.8%)			1 (2.8%)
Combined events				1 (2.8%)	1 (2.8%)		1 (2.8%)	3 (8.3%)
Middle distances					1 (2.8%)	1 (2.8%)	2 (5.6%)	4 (11.1%)
Long distances					4 (11.1%)		1 (2.8%)	5 (13.9%)
Marathon					1 (2.8%)		2 (5.6%)	3 (8.3%)
**Male athletes**		**1 (2.8%)**	**1 (2.8%)**		**2 (5.6%)**	**1 (2.8%)**	**9 (25.0%)**	**14 (38.9%)**
Sprints		1 (2.8%)			1 (2.8%)		1 (2.8%)	3 (8.3%)
Hurdles						1 (2.8%)		1 (2.8%)
Jumps					1 (2.8%)		1 (2.8%)	2 (5.6%)
Combined events			1 (2.8%)					1 (2.8%)
Middle distances							2 (5.6%)	2 (5.6%)
Long distances							1 (2.8%)	1 (2.8%)
Marathon							4 (11.1%)	4 (11.1%)

## Discussion

Across 24 top-level athletics championships, BSIs accounted for a small portion of all injuries (36 of 2,362; 1.5%). About half of the injuries were reported in endurance disciplines and the remaining injuries were reported in explosive disciplines. More than 90% of all BSIs occurred at the lower extremity, and mostly at the foot (50%) and the lower leg (33%). While a relatively uncommon form of overuse injury during international athletic championships, most athletes had significant lost time to training and competition as result of BSI.

It is well known that the risk of developing a BSI depends on the type of activity [1, 29]. Athletes participating in sports with impact and repetitive skeletal loading (e.g., road and track running, jumping, dancing) experience the highest risk of BSIs resulting from sport [9]. During international athletics championships, a small portion of 1.5% of all injuries were reported to be BSIs. By combining the data from 24 international competitions, this report expands on prior work describing in-competition injury profiles [16, 20–28]. The overall findings were similar to an injury surveillance study of the Olympic Games 2020 in Tokyo [12], but lower than in other studies on athletics athletes during the whole athletics season [7, 9, 30]. In a retrospective study among Swedish youth and adult athletic athletes, around 6% of all injuries were reported as BSIs [30]. A five-year prospective study of male youth athletics revealed that up to 20% of injuries in athletics may be result of BSIs [31]. However, only the minority of injuries (18 of 290) were classified as high-grade BSIs while most other BSIs were reported to be low-grade injuries. Varying definitions of BSIs across studies are a known problem, and differences in terminology influence the results of epidemiological studies. Therefore, an updated terminology is warranted [2]. Although speculative, several reasons may explain the overall low rate of BSIs during major athletic competitions. In athletics, the highest cumulative bone loading may be reached during training phases rather than competition phases. A study by Martinez-Silván et al. [31] reported that almost half of all injuries in male youth athletic athletes were recorded during the first four month (September to November) of a 12-month season. Typically, training intensity and volume are highest during pre-season or in the lead-up to a championship rather than during the competition phase. While the present study does not explore aspects of training contributing to BSI, it is well known that most athletes reduce training load prior to a major competition commonly referred to as tapering. Also, as compared to other forms of overuse injuries, BSIs mostly do not allow for participation in high-level competitions, and injured athletes may have not even travelled to the championships.

Although speculative, the athletes’ access to advanced imaging modalities prior, during or after a championship may bias reporting of injury rates. BSIs are typically diagnosed based on clinical history and examination with imaging studies being necessary to confirm diagnosis. While some athletes may prefer a diagnostic workup with their primary sports medicine physician, other athletes may have received BSI diagnosis at major competitions due to the ability to have greater access to advanced imaging. For BSIs, magnetic resonance imaging (MRI) is the diagnostic standard given its high sensitivity and specificity [5]. Freely available healthcare to all athletes regardless of their nationality is the standard of care during the Olympic Games and a strategic goal of the International Olympic Committee [12]. A lack of accessibility to MRI for some countries may contribute to inequitable care as demonstrated by a significant demand for MRI in the Olympic Village “Polyclinic” during the 2012 Olympic Games by low-income countries [32]. Given the injury burden of BSIs [6], all top-level events should aim for high-level medical care including access to MRI for all athletes. This is important as a delay of BSI diagnosis is often seen due to their subacute occurrence without a single traumatic event, and missing access to MRI may further delay the initiation of proper treatment [1]. Failure of early diagnosis may lead to a progression of injury, and a systematic review indicated an association between the MRI severity grading of the injury and the time needed to return to sports [5].

The present study revealed no significant differences in rate of BSI in female and male athletes. Prior work has shown that female athletes are at higher risk of developing a BSI compared to male athletes [1, 8]. In fact, a systematic review on running injuries demonstrated that BSIs and Achilles tendinopathies were running-related injuries with a statistically significant difference in sex-specific injury rate [33]. While Achilles tendinopathies occurred at a higher rate in male athletes, the female sex was associated with a higher risk of BSIs [33]. The present BSI rates of 2.0 and 0.9 per 1000 female and male athletes, although not statistically significant, are similar to previous epidemiolocal investigations demonstrating a 1.8-2.3-fold higher rate of BSI in females than in male athletes [1]. Various sex-specific risk factors may contribute to the predominance of BSI in women. Besides anatomical (e.g., cortex thickness) and biomechanical (e.g., coronal hip and knee peak angles) considerations, the influence of low energy availability state is seen as a contributing factor in the development of BSI [34]. The higher injury risk in female athletes has been at least partially explained by a higher prevalence of REDs (Relative Energy Deficiency in Sports) [34, 35]. The disruption of the hypothalamus-pituitary-gonadal axis may clinically demonstrate as amenorrhea/oligomenorrhea in female athletes, and a decrease in estrogen production, along with other hormones, is known to negatively affect bone health [36]. Consequently, athletes with REDs were shown to be at increased risk for low bone mineral density, impaired bone microarchitecture, and overall risk of developing a BSI [34]. Given that previous reports on BSI risk factors rarely address gender differences [3, 29], a more individualized approach could be an important strategy for injury prevention. In addition, it seems to be important that future studies investigate the epidemiological aspects of BSIs in the light of low energy availability and concerns for REDs.

Although sex and type of sports are known to influence the prevalence of BSIs, data on discipline-specific injury rates in athletics are scarce. Among all events, middle- and long-distance running accounted for 50% of BSIs, and these disciplines usually accounted for 25% of all registered athletes [15]. This finding is in line with previous epidemiological investigations. A retrospective 10-year study by Arendt et al. [37] identified distance runners accounting for most of the BSIs in athletic athletes. However, a twelve-month prospective study by Bennell et al. [7] showed no significant difference in overall injury rates between athletic disciplines but running was associated with a lower number of foot BSIs as compared to lower numbers of lower leg injuries in sprints, hurdles and jumps. Both the foot and lower leg are the two most common sites of injury as identified by a prospective 5-year study [38]. With 50% of BSIs located at the foot and 33% at the lower leg, our present results are in line with previous findings [7, 37, 39]. Nonetheless, a BSI may occur in any bone which sustains excessive loading in the presence of insufficient time for repair of bone microdamage [1, 7, 9]. For instance, the possibility of BSI of the upper extremities should be considered in throwing athletes [1], and notably a wrist BSI was reported by a combined event athlete.

We found a significant difference in BSI rate between outdoor and indoor championships. Potential explanations can be the shorter duration of the indoor championships. An indoor championship typically lasts 3–4 days as compared to 5–9 days for outdoor events, thereby decreasing the exposure to the BSI risk and the time to perform the diagnosis during the period of the championships. The shorter length of the running disciplines (the longest indoor event is 3000 m as compared to marathon for outdoor events) and the difference in the track geometry could be another explanation. Also, outdoor events (e.g. Olympics) are the highlight of the season for most athletes. Athletes who experience symptoms prior to competitions may have opted to skip indoor competitions in order to minimize further injury risk.

Most athletes had significant time loss as a result of BSI, and injuries were classified as severe injuries. However, six cases have been reported as no-time loss injuries. This reflects the wide spectrum of bone stress injuries encompassing low-grade injuries at low-risk injury sites to complete stress fractures at injury sites being prone to treatment complications [5, 6]. Of note, elite athletes may sometimes opt for management strategies that are not medically advised (e.g. participating in major competitions despite having pain).

Limitations of the injury surveillance of athletics championships have been previously discussed [19, 23]. Among others, diagnosis of injury was made by different clinicians, and reporting of injury was based on predefined categories. The numbers of registered athletes per disciplines were not available for Olympic Games, which does not allow us to perform comparison of the BSI incidence rates between disciplines. Only injuries that newly occurred during the championships were included. No statements can be made on injury rates prior to and after the championships. Furthermore, we did not have information on the portion of BSI that were confirmed using imaging. Also, small number of BSIs may limit ability to detect sex differences.

## Conclusions

The present study included 36 BSIs that accounted for 1.5% of all injuries during 24 top-level athletics championships. The majority of injuries occurred in the foot and the lower leg. BSI incidence was significantly higher during outdoor championships compared to indoor championships. Findings from this report can help clinicians with decision-making during athletic events and the development of preventive measures by gaining further insights into the injury rate and characteristics of BSIs.

## Data Availability

Data are not shared openly.
